# Adaptation and Validation of a Modified Broth Microdilution Method for Screening the Anti-Yeast Activity of Plant Phenolics in Apple and Orange Juice Models

**DOI:** 10.3390/life14080938

**Published:** 2024-07-26

**Authors:** Jan Staš, Marketa Houdkova, Jan Banout, Eduardo Duque-Dussán, Hynek Roubík, Ladislav Kokoska

**Affiliations:** 1 Department of Sustainable Technologies, Faculty of Tropical AgriSciences, Czech University of Life Sciences Prague, 165 00 Prague, Czech Republic; stas@ftz.czu.cz (J.S.); duque_dussan@ftz.czu.cz (E.D.-D.); 2 Department of Crop Sciences and Agroforestry, Faculty of Tropical AgriSciences, Czech University of Life Sciences Prague, 165 00 Prague, Czech Republic; houdkovam@ftz.czu.cz

**Keywords:** antifungal, food safety, natural preservatives, plant compounds, *Saccharomyces cerevisiae*, *Zygosaccharomyces*

## Abstract

Yeasts are the usual contaminants in fruit juices and other beverages, responsible for the decrease in the quality and shelf-life of such products. Preservatives are principally added to these beverages to enhance their shelf-life. With the increasing consumer concern towards chemical food additives, plant-derived antimicrobials have attracted the attention of researchers as efficient and safer anti-yeast agents. However, the methods currently used for determining their anti-yeast activity are time- and material-consuming. In this study, the anti-yeast effect of plant phenolic compounds in apple and orange juice food models using microtiter plates has been evaluated in order to validate the modified broth microdilution method for screening the antimicrobial activity of juice preservative agents. Among the twelve compounds tested, four showed a significant in vitro growth-inhibitory effect against all tested yeasts (*Saccharomyces cerevisiae*, *Zygosaccharomyces bailii*, and *Zygosaccharomyces rouxii*) in both orange and apple juices. The best results were obtained for pterostilbene in both juices with minimum inhibitory concentrations (MICs) ranging from 32 to 128 μg/mL. Other compounds, namely oxyresveratrol, piceatannol, and ferulic acid, exhibited moderate inhibitory effects with MICs of 256–512 μg/mL. Furthermore, the results indicated that differences in the chemical structures of the compounds tested significantly affected the level of yeast inhibition, whereas stilbenes with methoxy and hydroxy groups produced the strongest effect. Furthermore, the innovative assay developed in this study can be used for screening the anti-yeast activity of juice preservative agents because it saves preparatory and analysis time, laboratory supplies, and manpower in comparison to the methods commonly used.

## 1. Introduction

Juices obtained from fruits by the mechanical process are frequently consumed and appreciated worldwide, not just because of their attractive flavour characteristics and refreshing aspect. They are also considered a natural source of beneficial health components that contribute to human health, such as the variety of vitamins, minerals, and phytochemicals [1,2,3]. Many reports showed that approximately 20–40% of all fresh fruits are converted into juices [4,5,6]. This significant portion of fruit being processed into juice highlights their widespread popularity among consumers. The convenience and perceived health benefits of fruit juices have contributed to this high conversion rate. Additionally, advancements in technology and distribution have enabled the efficient production and widespread availability of fruit juices in various forms, including fresh-pressed, concentrated, and blended varieties [5]. Orange remains the dominant flavour, with a 43.8% share of the juice market, followed by apple with 16.9% of juice sales [7]. The popularity of orange juice can be attributed to its widespread availability, versatile flavour, and high levels of vitamin C. Apple juice, while not as dominant as orange juice, maintains a steady share due to its mild taste and perceived health benefits. As consumer preferences and health trends evolve, the dynamics of the juice market may continue to shift, creating opportunities for new and innovative fruit juice [8]. Due to the intrinsic properties of the juices, particularly their low pH value and low nitrogen and oxygen contents, fruit juices impose an adverse environment for most microorganisms. However, acid-tolerant bacteria and fungi (moulds and yeasts) use them as an ideal substrate for their growth. Yeast species, such as *Saccharomyces cerevisiae*, *Zygosaccharomyces bailii*, and *Zygosaccharomyces rouxii*, are among the most concerning microbial contaminants and are well known for their potential to deteriorate juices and other beverages [9]. They pose considerable challenges to the fruit juice industry, and the production of safe and high-quality products requires careful monitoring and application of control measures [10,11]. These yeasts, which pose a potential risk to the consumers [12,13], typically come from microbiota, which are usually present on the surfaces or inside the fruits that were contaminated due to inappropriate harvest and postharvest manipulation, storage conditions and distribution [14].

Juice preservation techniques aim to inhibit the growth of microorganisms, enzymes, and chemical reactions that can lead to spoilage. By reducing the microbial load and preventing enzymatic browning and oxidation, the quality and safety of the juice can be prolonged. Preservation is achieved through various methods, including pasteurization, canning, freezing, freeze-drying, and the addition of preservatives. Each preservation method has specific advantages and limitations in terms of its impact on the nutritional composition and sensory attributes of the juice. Moreover, it is essential to consider the potential effects of preservation techniques on the overall nutritional value of the juice and its impact on consumers. In short, the main reason for preserving juices is to prevent spoilage while maintaining nutritional and sensory quality [15]. Chemical preservatives such as benzoic and sorbic acids, potassium sorbate, sodium benzoate, and sulphites are commonly used to extend the shelf-life of juices and beverages as an alternative to physical methods, which are considered too energy-consuming or costly [16,17,18] and can lead to losses of nutrients and changes in physicochemical and organoleptic properties [19,20,21]. Sulphites, encompassing various forms such as sulphur dioxide, bisulphites, or metabisulfites, are extensively employed in the food industry. Their primary purpose lies in controlling the proliferation of undesirable microorganisms, thereby ensuring the preservation of fruits, fruit juices, wines, and numerous other food products. This widespread use is attributed to their efficacy in inhibiting microbial growth, safeguarding against spoilage, and extending the shelf-life of these perishable food items. However, the adverse effects on health (e.g., allergies, asthma, diarrhoea, headache, nausea, palpitations, and cancer) caused by these preservatives were reported in several studies [22,23,24,25,26]. Therefore, the growing global inclination towards eliminating sulphites as preservative agents stems from an increasing awareness of potential health concerns associated with their consumption. As consumers prioritise natural and minimally processed food options, the food industry is witnessing a shift towards alternative preservation methods that meet both the demand for safety and the desire for clean-label products [27]. As a result of this effort, the interest in the use of natural substances to prevent un-pasteurized juices from microbiological spoilage has increased significantly in recent years. This interest arises due to the high demand for healthy, fresh-like, and safe foods that contain as few preservatives as possible while assuring their safety and quality characteristics [18,28,29]. Moreover, the increasing resistance of juice spoilage yeasts to common antimicrobials has also been observed [30,31,32], which supports the need to search for new antifungal agents. Plant-derived antimicrobials, such as essential oils, plant extracts, organic acids, and phenolic compounds, have shown their feasibility for use in some food products [33,34,35,36,37]. In addition to their antimicrobial properties, plant-derived compounds often provide functional and sensory benefits, such as flavour enhancement and antioxidant activity. However, it is important to note that the application of these natural antimicrobials requires the careful consideration of factors such as concentration, compatibility with other ingredients, and regulatory requirements to ensure their effectiveness and safety in food products. Several studies have confirmed that certain classes of phenolic compounds, such as flavonoids, phenolic acids, phenylpropanoids, quinones, stilbenes, and tannins have great potential to inhibit the growth of yeasts that cause the deterioration of food and beverages [38,39,40]. Among antifungal phenolics, stilbenes produced significant effects against various species of food-pathogenic yeasts such as *Dekkera bruxellensis*, *Hanseniaspora uvarum*, *Saccharomyces cerevisiae*, *Zygosaccharomyces bailii*, and *Zygosaccharomyces rouxii* [41,42,43,44,45].

During the last few decades, specific laboratory methods for detecting the antimicrobial activity of compounds and extracts in juice food models have been developed. However, many of them are time-, labour-, and material-consuming. In addition, they are unsuitable for high-throughput screening and, therefore, do not allow a researcher to identify rapidly active antimicrobial agents with the help of automation. A prevalent challenge encountered in antimicrobial testing within the realm of juice analysis is the substantial volume of juice necessitated for evaluation, often ranging between 1 and 100 mL per sample concentration. This high amount in required juice volume per sample concentration poses practical constraints, as it demands notable quantities of juice for each testing iteration [46,47,48]. Moreover, attaining the requisite sterility of juice typically involves subjecting it to prolonged heat treatment, often exceeding 15 min at temperatures surpassing 100 °C [49,50,51]. While this thermal process effectively eliminates microbial contaminants, it unavoidably induces alterations in the structural composition and various inherent properties of the juice [44]. Such extended exposure to high temperatures can lead to changes in flavour profiles, nutrient content, colour, and overall physicochemical attributes, thereby impacting the sensory and nutritional qualities of the juice [50,52,53,54]. The standard broth microdilution assay is recommended by the Clinical and Laboratory Standards Institute (CLSI) for the screening of antimicrobial agents due to the high-throughput potential, considerable savings in materials usage, and the requirement of a small amount of sample; moreover, this method could be applied for different microorganisms [55,56,57]. Despite these advantages, the broth microdilution method is minimally used by researchers in food model studies. To the best of our knowledge, so far, only one recent study using microplates for testing the antifungal activity of natural agents in the juice model has been published [58].

Our study has two simultaneous objectives: (1) to adapt and validate a modified broth microdilution method for assessing anti-yeast activity in juice models and (2) to evaluate the anti-yeast activity of specific plant phenolics using this method in apple and orange juice food models using microtiter plates.

## 2. Materials and Methods

### 2.1. Chemicals

Curcumin (purity >94%, CAS 458-37-7), chlorogenic acid (>95%, 327-97-9), eriodyctiol (>95%, 4049-38-1), ferulic acid (>99%, 537-98-4), luteolin (>98%, 491-70-3), myricetin (>96%, 529-44-2), naringenin (>99%, 67604-48-2), oxyresveratrol (>95%, 29700-22-9), phlorizin (>99%, 60-81-1), piceatannol (>98%, 10083-24-6), pterostilbene (>98%, 537-42-8), resveratrol (>99%, 501-36-0), and sodium metabisulfite (>99%, 7681-57-4) were obtained from Sigma-Aldrich (Prague, Czech Republic). Dimethyl sulfoxide (DMSO) (Penta, Praha, Czech Republic) was used as a solvent for the compounds tested.

### 2.2. Yeast Strains and Growth Media

The anti-yeast activity was evaluated against *Saccharomyces cerevisiae* (DSM 2548) purchased from the German Collection of Microorganisms and Cell Cultures (Braunschweig, Germany) and two *Zygosaccharomyces* species obtained from the Czech Collection of Microorganisms (Brno, Czech Republic), namely *Z. bailii* (CCM 8239) and *Z. rouxii* (CCM 8224). The sub-cultures were maintained on Sabouraud Dextrose agar slants (*S. cerevisiae*) and gelatine disks (*Z. bailii*, *Z. rouxii*) at 4 °C. The inocula of tested yeasts were grown in Sabouraud Dextrose broth (*S. cerevisiae*) and Glucose Yeast Peptone broth (*Z. bailii*, *Z. rouxii*) at 25 °C for 48 h. All the cultivation media were obtained from Oxoid (Basingstoke, UK).

### 2.3. Preparation of Juices

The juices were prepared by squeezing fresh orange (*Citrus sinensis*) and apple (*Malus domestica*) fruits, which were checked beforehand to exclude the rotten, cracked, or unripe ones and washed several times thoroughly to remove unwanted dirt. The fruits were obtained from the local market (Eso Potraviny, Prague, Czech Republic), washed with distilled water, and peeled with a sterile knife. To separate the fruit pulp, the juice was filtered through a kitchen iron sieve with a pore size of 0.5 mm and subsequently centrifuged at room temperature using a Minispin centrifuge (Eppendorf, Hamburg, Germany) at 120 rpm for 2 min. The liquid part was then transferred to sterile plastic Falcon™ 50 mL High-Clarity Conical Centrifuge Tubes (Gama Group, Ceske Budejovice, Czech Republic), stored at −25 °C and used within 5 days [59]. Before the testing, the juice was sterilised by filtration through a Pragopor membrane nano filter (Pragochema, Uhrineves, Czech Republic) with a pore size of 0.23 μm in a glass vacuum filtration system (Sartorius, Gottingen, Germany). From this second filtration, sterile transparent juice was obtained and directly used. The pH of the juices measured by a CyberScan pH 510 (Eutech Instruments, Singapore) varied between 3.3 and 3.6 for apple juice and 3.5 and 3.7 for orange juice.

### 2.4. Anti-Yeast Assay

The anti-yeast activity was determined by the broth microdilution method performed in standard 96-well microtiter plates (Gama group, well volume = 400 μL) according to the Clinical and Laboratory Standards Institute [60], European Committee on Antimicrobial Susceptibility Testing [61], Food and Drug Administration [62], and International Organization for Standardization [63], modified for the simulation of conditions of yeast growth in a juice matrix. Instead of broth, 100 μL of sterile juice was used as a medium in each well. Each sample of tested compound was dissolved in DMSO, except for sodium metabisulfite, for which the deionised water was used. All the samples were then diluted in sterile juices. Eight two-fold serially diluted concentrations of samples in a range of 8–1024 μg/mL were prepared and inoculated with 5 μL of yeast suspension with a concentration of 10^7^ CFU/mL. The inoculated and non-inoculated juice wells were prepared as growth and purity controls, respectively. After the inoculation, microtiter plates were incubated at 25 °C for 24 h in the Peltier-cooled incubator IPP55 (Memmert, Buechenbach, Germany), and yeast growth was then measured spectrophotometrically as turbidity using a Cytation 3 Multimode Reader (BioTek Instruments, Winooski, VT, USA) at 405 nm [64]. The minimum inhibitory concentrations (MICs) were expressed as the lowest concentrations, showing at least a ≥80% reduction of microorganisms’ growth compared to the compound-free growth control. The assay was performed as three independent experiments, each carried out in triplicate, and median/modal values were used for final MIC determination. According to the widely accepted standard in MIC testing, the mode and median were used for the final value calculation when the triplicate endpoints were within the two- and three-dilution range, respectively. Sodium metabisulfite was used as the positive reference control in a range of concentrations 8–1024 μg/mL. As a result of experiments performed without dissolved compounds, DMSO and distilled water did not inhibit the yeast growth of any strain at the tested concentrations (≤1%).

### 2.5. Statistical Analysis

The statistical analysis was conducted for each treatment, meaning that the microbial growth-inhibitory effect of all individual compounds on each of the yeasts was evaluated in both juices, taking the evaluated MIC in juices as the response variable. All data were analysed through a variance analysis (ANOVA—one way). Also, probability values of *p* < 0.05 were considered significant. The difference between the treatment means was estimated using multiple ranges of Tukey’s HSD (honestly significant difference) with a 95% confidence interval test using the R software (version 4.3.2).

At the same time, a principal component analysis (PCA) was carried out for interpreting results as it simplifies complex datasets. By transforming original variables into uncorrelated components, PCA reveals underlying patterns that are not readily apparent in raw data. It condenses information, aiding in visualization and identifying influential factors. Ordering components by variance explains which variables, like MIC, yeast strains, or juice types, contribute most to observed differences. This facilitates uncovering hidden structures, detecting outliers, making informed decisions, and enhancing data interpretation and analysis.

## 3. Results and Discussion

Four of the twelve compounds tested in this study exhibited inhibitory activity against all three yeasts grown in both juices. As it can be seen in Table 1, the level of the inhibitory effect depended significantly on differences in the chemical structures of tested compounds. Stilbenes with methoxy and hydroxy groups (pterostilbene) or four hydroxy groups (oxyresveratrol and piceatannol) and phenolic acid (ferulate) with methoxy and hydroxy groups produced the growth-inhibitory effect against all yeasts tested. Pterostilbene, a dimethylated analogue of resveratrol with methoxy groups at positions A-3, -5 and hydroxyl group on B-4′, demonstrated the most potent anti-yeast effect within all yeasts tested. Principally, the presence of methylated hydroxyphenyl groups in the pterostilbene structure is acknowledged to raise its biological activity [42,65]. Moreover, the results indicated that the higher number of hydroxyl groups in the compound structure strengthens its biological activity. This follows the evidence that the increased hydroxylation of phenolic compounds leads to higher toxicity to microorganisms [66]. All the flavonoids and other tested compounds did not show any antifungal activity, which is contrary to the study in [42], in which the flavonoids inhibited the growth of *S. cerevisiae, Z. bailii,* and *Z. rouxii* at MICs varying from 256 to 512 μg/mL. Various results of yeast susceptibility testing can be caused by a modification of our assay, especially by the difference between the composition of the fruit juices and the standard growth medium, which corresponds with the study of [67], which observed that different compositions of growth media affected the anti-yeast activity of tested agents. Similarly, the study of [68] reported the effect of different growth media on the antimicrobial efficacy of compounds tested against *Candida* species.

Among the individual compounds assayed, the greatest anti-yeast action was observed for pterostilbene in both orange and apple juices against all microorganisms tested, with MIC values ranging from 32 to 128 μg/mL, followed by piceatannol (MICs = 256–512 μg/mL) and oxyresveratrol (MICs = 512–1024 μg/mL). These results are in accordance with several studies that proved the anti-yeast activity of these three stilbenes. For example, pterostilbene, piceatannol, and oxyresveratrol have a high growth-inhibitory effect against *C. tropicalis* [43,69,70]. Moreover, these results were confirmed by the study, where a newly developed in vitro tetrazolium-based colorimetric assay using standard 96-well microtiter plates and MTT [3-(4,5-dimethylthiazol-2-yl)-2,5-diphenyltetrazolium bromide] was used for the high-throughput screening of the anti-yeast activity of plant-derived preservative candidates in juice food models [71]. Despite the fact that the anti-yeast activity of these compounds is known in the literature, to the best of our knowledge, this is the first report on their inhibitory effect on the growth of pathogenic yeasts in fruit juice. These results suggest that stilbenes could be used as perspective agents to control the growth of beverage-spoilage microorganisms. Nevertheless, further research should explore cost-effective and scalable solutions. Our study used only pure compounds (with a purity higher than 94%), which are more expensive than plant extracts with lower purity and less suitable for large-scale production [72,73]. Especially among the stilbenes, pterostilbene emerges as an especially promising candidate for beverage preservation. This compound, renowned for its antioxidant, anti-inflammatory, and anticarcinogenic properties [74,75] naturally occurring in *Vitis vinifera* and *Vaccinium corymbosum* [76,77], seems to be the most promising agent for not only extending shelf-life but also potentially enhancing the health-related aspects of preserved beverages. Piceatannol, a hydroxylated analogue of resveratrol naturally occurring in various plants such as *V. vinifera* and *Passiflora edulis*, is known for exhibiting strong antimicrobial and antioxidative activity as well as anticancer potential [78,79,80]. Furthermore, the results showed that pterostilbene’s antimicrobial action is significantly higher compared to oxyresveratrol, another stilbene that naturally occurs in *V. vinifera* and *Morus alba* [81]. In addition to the stilbenes, ferulic acid was the only compound showing anti-yeast activity with MICs ranging from 512 to 1024 μg/mL. These values are slightly lower than in the study [82] in which ferulic acid exhibited an MIC = 2000 μg/mL against *S. cerevisiae* in the malt-extract growth medium when assayed by the standard microplate method. Most microorganisms tested were more susceptible to active phenolics (MICs ≥32 μg/mL) than to sodium metabisulphite (MICs ≥512 μg/mL), which corresponds with previously published studies [42,83]. The standard deviations of yeast-growth control wells were as follows: for apple juice, *S. cerevisiae* (SD = 0.088), *Z. bailii* (SD = 0.064), and *Z. rouxii* (SD = 0.038); and for orange juice, *S. cerevisiae* (SD = 0.125), *Z. bailii* (SD = 0.066), and *Z. rouxii* (SD = 0.069). These results indicate that the variations in individual yeast growth were minimal, ensuring that the juice matrix provided a stable environment for consistent and reliable MIC testing. The values of MICs determined for active plant phenolic compounds in both juice food models are shown in Table 2.

Compared to other in vitro quantitative methods assaying antimicrobial agents in fruit juices using macrodilution in tubes and flasks [84,85], microplate-based methods allow for the cost- and labour-effective high-throughput screening of antimicrobials in low volumes of growing matrices. As discussed in several studies, they are suitable for the simple and fast determination of the antimicrobial potential of beverage preservatives at different concentrations, while several agents may be assessed in one microplate [86,87,88,89,90]. Our study introduces a novel modified broth microdilution method that further enhances these advantages by being not only more cost- and labour-effective but also significantly more rapid than traditional methods. This method allows for the quicker determination of antimicrobial activity, making it an ideal tool for high-throughput screening. Recently, a published study by Wang and Sun [58] used microplates for testing the antifungal activity of natural agents in apple juice sterilised at 100 °C for 30 min. Consistent with their results, our study shows that the susceptibility testing of food pathogenic yeasts to plant-derived compounds using a juice food model based on the modified broth microdilution method provides accurate and reproducible results. In addition, our experiments proved the suitability of juice sterilisation by membrane filtration. Heat treatment such as pasteurisation at high temperatures for a long time (80~100 °C, 10–30 min) can inevitably impact the sensory characteristics, nutritional integrity, and, specifically, the vitamin C content of the juice. Cumulatively, these effects contribute to a reduction in the overall quality of the product. This reduction in quality emphasises the need for alternative sterilisation methods that can maintain the organoleptic and nutritional properties of the juice while ensuring microbial safety [91,92,93].

In the case of the activity of phenolic compounds in orange juice, a significant statistical difference was obtained between the means of the treatments. This indicates that there is a difference between the inhibitory effect of the compounds against the yeasts (*S. cerevisiae*, *Z. bailii*, and *Z. rouxii*).

Concerning the multiple-range test, it was seen that the inhibition levels presented by the pterostilbene treatment and sodium metabisulfite (control) are significantly different, and the same behaviour was seen when comparing the inhibition levels of pterostilbene and ferulic acid. Both groups had a confidence level of under 95%; this indicates that when observing the MIC values of the other evaluated compounds, they do not display a statistical difference in their growth-inhibitory effect against the yeasts. The pterostilbene exhibited the lowest MIC values and demonstrated the least variability in the data. This suggests that the inhibitory effect of pterostilbene on the yeasts was suitable and consistently homogeneous. The consistent behaviour observed in the data indicates pterostilbene’s more reliable and predictable inhibition performance than other compounds.

On the other hand, in the case of compounds studied in apple juice, a significant difference between the MIC values (*p* = 0.0498) between the treatments evaluated was also obtained. After evaluating the multiple-range values, a significant difference was recorded between pterostilbene and oxyresveratrol and between pterostilbene and sodium metabisulfite.

When examining the inhibitory effect of the compounds in apple juice, pterostilbene demonstrated analogous outcomes to those observed in orange juice. The achievement of a notably low MIC, coupled with minimal deviations concurrent with a high degree of uniformity, underscores its efficacy and homogeneous behaviour. The congruence in results between the two fruit juices suggests a consistent and robust inhibitory profile of pterostilbene. Its performance in apple juice mirrors the established trend noted in orange juice, reinforcing the compound’s potential applicability in various juices. Non-significant comparisons are not displayed due to their lack of relevance.

Pterostilbene is the dimethylated analogue form of resveratrol; it has been reported that it may be more effective in preventing microbial growth [43,94]. This seems to be due to pterostilbene’s methoxy residue, which plays a crucial role in its antifungal activity [43]. It has been reported that the method of action of this stilbene, having two methoxy residues, one at each end of the molecule, allows it to have non-ionic surfactant-like characteristics, which causes damage to the lipid bilayer, causing an imbalance in the permeability of the plasma membrane—in this case, of the yeast *S. cerevisiae* [95]. This mechanism of action may also be applicable to the other two yeasts investigated in this study.

**Figure 1:**
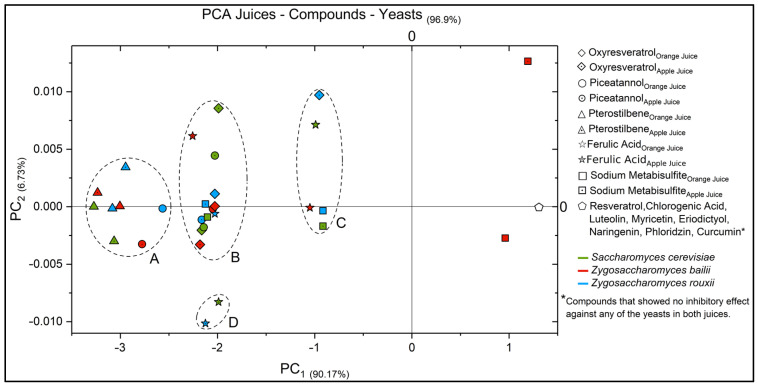
Principal component analysis (PCA) of the evaluated compounds and their yeast inhibitory behaviour per juice.

Figure 1 displays the outcome of the PCA for each treatment, where each axis (x–y) captures a percentage of the original data variability—the PC_1_ captured 90.17%, whereas the PC_2_ depicts 6.73%; therefore, both axes captured 96.9% of the total variance in the data.

Points that are close to each other exhibit similar yeast growth-inhibitory activity; however, four groups were identified and clustered in the plot: the first one (Group A) consists of pterostilbene, which belongs to the chemical structure of stilbenes. This compound exhibited a similar growth-inhibitory effect for the three evaluated yeasts in orange and apple juice, highlighting that this compound also showed the lowest MIC, disregarding the yeast and juice type. Group A also comprises piceatannol in orange and apple juice regarding the inhibition action against *Z. bailli* and *Z. rouxii,* respectively.

The second group (Group B) comprises oxyresveratrol in all tests except for *Z. rouxii*/apple juice; piceatannol for all scenarios except *Z. rouxii*/orange juice and *Z. bailii*/apple juice; ferulic acid for *Z. rouxii*/orange juice and *Z. bailii*/apple juice; and sodium metabisulfite for *S. cerevisiae*/orange juice and *Z. rouxii*/apple juice. Stilbenes and phenolic acids represent Group B. Similar inhibitory effects were achieved for this group as the clustered components shared similar MIC values; nevertheless, the inhibition of the growth of all three yeasts was seen only in apple juice.

Group C, represented by oxyresveratrol for *Z. rouxii*/apple juice; ferulic acid for *S. cerevisiae*/orange juice and *Z. bailii*/orange juice; and sodium metabisulfite with very similar behaviour for *Z. rouxii*/orange juice and *S. cerevisiae*/apple juice, displayed a related inhibitory effect; however, the growth inhibition of the three yeasts was achieved only in orange juice for this group.

Lastly, a sub-cluster labelled D was discovered for ferulic acid inhibiting *S. cerevisiae* and *Z. bailii*, denoting a specific effect when controlling their growth in apple juice.

Finally, in this study, the compounds resveratrol and chlorogenic acid, all evaluated flavonoids, and diarylheptanoids, which lack anti-yeast activity, were located on axis 0 of the graph (pentagon).

## 4. Conclusions

The results of a series of experiments with plant-derived phenolic compounds confirmed the effectiveness of the modified broth microdilution method for assessing the anti-yeast activity of natural agents in fruit juices. Therefore, it can be concluded that this innovative assay can be used to filter the anti-yeast activity of juice preservative agents because it saves time, material, and labour compared to the methods usually used. This novel modified broth microdilution method is not only cost- and labour-effective but also rapid, significantly reducing the time required for antimicrobial testing. Among all compounds tested, four showed significant inhibitory activity against all yeasts tested in both orange and apple juices, while pterostilbene, followed by oxyresveratrol, piceatannol, and ferulic acid, produced the strongest anti-yeast effects. The level of inhibition depended significantly on differences in the chemical structures of tested compounds, whereas stilbenes produced the strongest anti-yeast effect. In summary, stilbenes, especially pterostilbene, were shown to be worthy of attention and promising agents for further investigation in the field of beverage additives. In conclusion, only the stilbene group consistently demonstrated homogeneity in its inhibitory activity against all three yeast strains, regardless of the juice type. However, more detailed research on their technological and organoleptic properties is necessary before any practical application.

## Figures and Tables

**Table 1:** Chemical structures and anti-yeast activity of natural compounds assayed in this study.

Chemical Structure	R^1^	R^2^	R^3^	R^4^	R^5^	Name	Anti-Yeast Activity
Stilbenes							
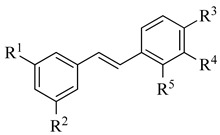	OH	OH	OH	H	OH	oxyreseveratrol	Yes
	OH	OH	OH	OH	H	piceatannol	Yes
	OCH_3_	OCH_3_	OH	H	H	pterostilbene	Yes
	OH	OH	OH	H	H	resveratrol	No
Phenolic acids							
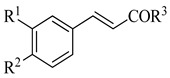	OCH_3_	OH	OH	-	-	ferulic acid	Yes
	OH	OH	quinic acid	-	-	chlorogenic acid	No
Flavonoids							
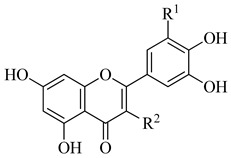	OH	H	-	-	-	luteolin	No
	H	OH	-	-	-	myricetin	No
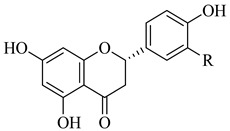	OH	-	-	-	-	eriodictyol	No
	H	-	-	-	-	naringenin	No
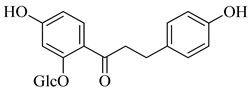	-	-	-	-	-	phloridzin	No
Diarylheptanoids							
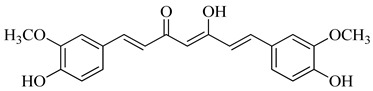	-	-	-	-	-	curcumin ^a^	No

^a^ Shown in the keto-enol form, which is more abundant in nature than the diketo form.

**Table 2:** The growth-inhibitory effect of plant phenolic compounds active against yeasts in apple and orange juice food models.

Compound	Yeast/Juice Food Model/MIC ^a^ (μg/mL)					
	*Saccharomyces cerevisiae*		*Zygosaccharomyces bailii*		*Zygosaccharomyces rouxii*	
	Orange	Apple	Orange	Apple	Orange	Apple
Ferulic acid	1024	512	1024	512	512	512
Oxyresveratrol	512	512	512	512	512	1024
Piceatannol	512	512	512	256	256	512
Pterostilbene	32	32	32	64	32	128
Sodium metabisulfite ^b^	512	1024	>1024	>1024	1024	512

^a^ MIC: minimum inhibitory concentration. ^b^ Positive reference control.

## Data Availability

Data are contained within the article.
